# Excited-State Dynamics of the Thiopurine Prodrug 6-Thioguanine: Can N9-Glycosylation Affect Its Phototoxic Activity?

**DOI:** 10.3390/molecules22030379

**Published:** 2017-02-28

**Authors:** Brennan Ashwood, Steffen Jockusch, Carlos E. Crespo-Hernández

**Affiliations:** 1 Department of Chemistry and Center of Chemical Dynamics, Case Western Reserve University, Cleveland, OH 44106, USA; bga11@case.edu; 2 Department of Chemistry, Columbia University, New York, NY 10027, USA; sj67@columbia.edu

**Keywords:** sulfur-substituted DNA bases, prodrugs, phototoxicity, excited-state dynamics, singlet oxygen generation, glycosylation, low-temperature emission, transient absorption, TD-DFT

## Abstract

6-Thioguanine, an immunosuppressant and anticancer prodrug, has been shown to induce DNA damage and cell death following exposure to UVA radiation. Its metabolite, 6-thioguanosine, plays a major role in the prodrug’s overall photoreactivity. However, 6-thioguanine itself has proven to be cytotoxic following UVA irradiation, warranting further investigation into its excited-state dynamics. In this contribution, the excited-state dynamics and photochemical properties of 6-thioguanine are studied in aqueous solution following UVA excitation at 345 nm in order to provide mechanistic insight regarding its photochemical reactivity and to scrutinize whether N9-glycosylation modulates its phototoxicity in solution. The experimental results are complemented with time-dependent density functional calculations that include solvent dielectric effects by means of a reaction-field solvation model. UVA excitation results in the initial population of the S_2_(ππ*) state, which is followed by ultrafast internal conversion to the S_1_(nπ*) state and then intersystem crossing to the triplet manifold within 560 ± 60 fs. A small fraction (ca. 25%) of the population that reaches the S_1_(nπ*) state repopulates the ground state. The T_1_(ππ*) state decays to the ground state in 1.4 ± 0.2 μs under N_2_-purged conditions, using a 0.2 mM concentration of 6-thioguanine, or it can sensitize singlet oxygen in 0.21 ± 0.02 and 0.23 ± 0.02 yields in air- and O_2_-saturated solution, respectively. This demonstrates the efficacy of 6-thioguanine to act as a Type II photosensitizer. N9-glycosylation increases the rate of intersystem crossing from the singlet to triplet manifold, as well as from the T_1_(ππ*) state to the ground state, which lead to a ca. 40% decrease in the singlet oxygen yield under air-saturated conditions. Enhanced vibronic coupling between the singlet and triplet manifolds due to a higher density of vibrational states is proposed to be responsible for the observed increase in the rates of intersystem crossing in 6-thioguanine upon N9-glycosylation.

## 1. Introduction

Sulfur-substituted purine nucleobases have long been prescribed to treat inflammatory bowel disease, arthritis, and various cancers [1,2,3,4,5,6]. Each act as a prodrug, in which their active form is produced following cellular metabolization. It has been shown that prolonged treatment of patients with these thiopurines is associated with a 10-fold and 65- to 250-fold increase in basal and squamous cell carcinomas, respectively [3,5]. The drastic increase in skin cancers is thought to result primarily from the metabolization of the thiopurine prodrugs into cellular DNA followed by photosensitized damage upon UVA absorption. 

**Figure 1:**
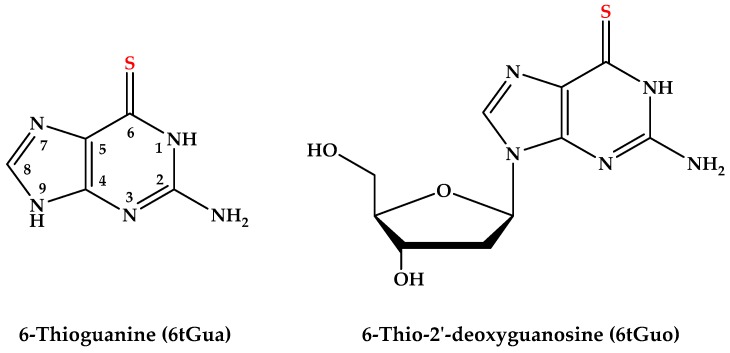
Molecular structures and common ring numbering of the nucleobase chromophore.

6-Thioguanosine (6tGuo) is produced and incorporated into DNA following extensive metabolization of 6-thioguanine (6tGua), 6-mercaptopurine, or azathiopurine [7,8,9,10,11]. 6tGuo has been shown to induce DNA damage and cell death upon exposure to UVA radiation, which is correlated to the significant increase in skin cancers following prolonged treatment with thiopurine prodrugs [9,11,12,13,14,15,16,17,18]. It is thought that 6tGuo may photosensitize cells through a Type II mechanism due to its relatively high singlet oxygen (^1^O_2_) yield of ca. 25% [19] and more efficient light absorption compared to that for the canonical base guanine in aqueous solution [19,20]. This hypothesis is supported by the observation of O_2_-dependent generation of the photoproducts guanine-6-sulfinate and guanine-6-sulfonate [13,15,21,22]. However, other studies indicate that 6tGuo also reacts directly with biological substrates, forming DNA intra- and inter-strand crosslinks and DNA-protein crosslinks [17,23,24,25]. The precursor and main chromophore of 6tGuo, 6tGua (see Figure 1), is also known to induce significant cell death following UVA irradiation [11], showing that 6tGua itself contributes to the overall photocytotoxic activity of the prodrug. Complete elucidation of the underlying photophysics of 6tGua and 6tGuo is required in order to understand their phototoxicity in cellular environments. Recently, 6tGuo was shown to undergo ultrafast intersystem crossing (ISC) following UVA excitation to populate a long-lived triplet state [26], which has been proposed to be responsible for direct reactions with biological substrates, as well as the production of reactive oxygen species (ROS) [17,19,23,24,25]. 

While the only structural difference between 6tGua and 6tGuo is N9-glycosidic substitution (Figure 1), recent work has shown that the sugar substituent can play a significant role in the excited-state properties of sulfur-substituted DNA bases [27,28]. Taras-Goślińska et al. showed that N1-glycosylation of the thiopyrimidine 2-thiothymine reduces the lifetime of the lowest-energy triplet state, which potentially reduces its reactivity towards DNA [27]. Additionally, Pollum et al. found that 2-thiothymidine undergoes intersystem crossing (ISC) more rapidly than 2-thiothymine [28], but it has yet to be shown whether similar changes occur in the thiopurine prodrugs upon N9-glycosylation. In particular, the role that glycosylation has on the photochemistry and phototoxicity of 6tGua is of great relevance due to its wide clinical applications and the reported increased incidence of skin cancer among patients with prolonged treatment [3,5]. In order to fill this knowledge gap, we have investigated the excited-state dynamics of 6tGua from femtoseconds to microsecond in aqueous solution, its reactivity toward molecular oxygen, and compared the results with those performed previously for 6tGuo under identical conditions. In addition, the time-dependent implementation of density functional theory (TD-DFT) is used to compute vertical excitation energies for 6tGua in aqueous environment and the results are compared to those previously reported for 6tGuo under equal conditions.

## 2. Results and Discussion

### 2.1. Steady-State Absorption of 6tGua

**Figure 2:**
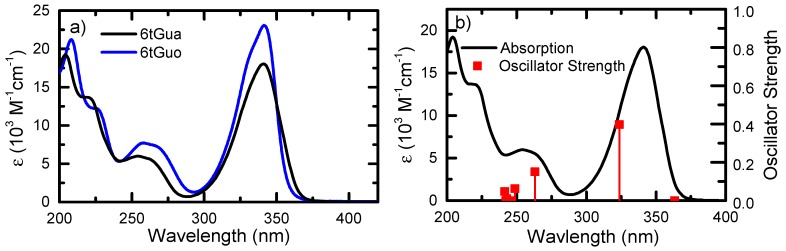
(**a**) Ground-state absorptivity of 6tGua and 6tGuo in PBS pH 7.4; (**b**) Ground-state absorptivity spectrum of 6tGua in PBS plotted with oscillator strengths calculated at the TD-PBE0/IEFPCM/6-311++G(d,p) level of theory. Vertical excitation energies were redshifted by 0.15 eV relative to those reported in Table 1 to better match the experimental absorptivity spectrum.

Figure 2 shows the ground-state absorptivity spectra of 6tGua and 6tGuo in phosphate buffer saline (PBS) solution at pH 7.4. The lowest-energy absorption band of 6tGua has a maximum at 341 nm (18,000 M^−1^cm^−1^). UVC maxima are observed at 254 nm (6000 M^−1^cm^−1^) and 204 nm (19,200 M^−1^cm^−1^) with a shoulder at 220 nm (13,500 M^−1^cm^−1^). 6tGuo has a very similar ground-state absorption spectrum, showing maxima at 341 nm (23,000 M^−1^cm^−1^), 258 nm (7700 M^−1^cm^−1^), and 208 nm (21,100 M^−1^cm^−1^) with a shoulder at 227 nm (12,000 M^−1^cm^−1^). N9-glycosylation increases the absorptivity coefficients of both the UVC and UVA absorption bands.

### 2.2. Steady-State and Time-Resolved Emission of 6tGua

**Figure 3:**
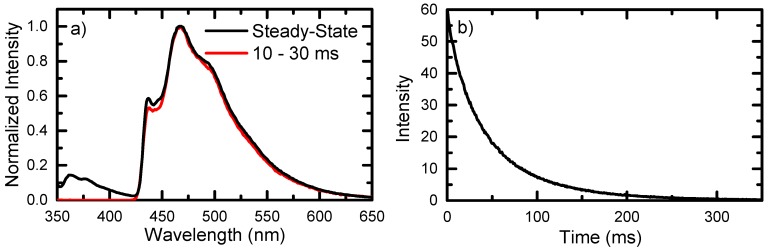
(**a**) Emission spectra of 6tGua in Tris buffer pH 7.4 in frozen matrix at 77 K following steady-state (black) and pulsed (red) excitation at 340 nm. The time-resolved spectrum was recorded 10–30 ms following pulsed excitation; (**b**) Representative phosphorescence decay trace of 6tGua monitored at 480 nm following pulsed excitation at 340 nm in frozen matrix at 77 K.

At room temperature, 6tGua shows only weak luminescence that is dominated by fluorescence from the photoproduct guanine-6-sulfonate (see Supporting Information) [29]. In a frozen matrix of Tris buffer pH 7.4 at 77 K, luminescence from 6tGua was observed. Under steady-state conditions following 340 nm excitation, a strong emission band is observed from 430 nm to 550 nm, showing a maximum at 468 nm and shoulders at 437 nm and 494 nm. A weaker emission band is also observed from 350 nm to 400 nm, exhibiting maxima at 362 nm and 377 nm. When using pulsed 340 nm excitation and time-gated detection (10–30 ms), only the luminescence band from 430 nm to 550 nm is observed (Figure 3a), which decays with a lifetime of 45 ms (Figure 3b). Due to its long lifetime, significant redshift of 62 nm (3780 cm^−1^) from the lowest-energy absorption (λ_max_), and quenching by molecular oxygen at room temperature (Figure S2), the emission band from 430 nm to 550 nm is assigned to phosphorescence emission from the lowest-energy triplet state to the ground state of 6tGua. This assignment is in agreement with previous emission spectra of 6tGua [30,31,32,33], specifically those reported at pH > 7 [30], and has been observed in several thionyl-containing aromatic compounds [34,35,36,37,38]. A triplet energy of 2.84 eV was determined from the highest-energy maximum of the emission band (437 nm), which is close in energy with vertical (2.73 eV) and adiabatic (3.04 eV) T_1_ state energy computed from TD-DFT calculations (see below). While a lifetime of 45 ms may be short for phosphorescence from a ππ* state in aromatic carbonyl compounds [39,40], the enhanced spin-orbit coupling following sulfur-substitution is expected to reduce the magnitude of the lifetime of phosphorescence, as observed previously for other sulfur-substituted DNA bases [41,42]. The weaker luminescence band from 350 nm to 400 nm overlaps the ground-state absorption and decays within the time resolution of the time-gated detection, suggesting that it is due to fluorescence emission from 6tGua.

### 2.3. Quantum-Chemical Calculations of 6tGua

**Figure 4:**
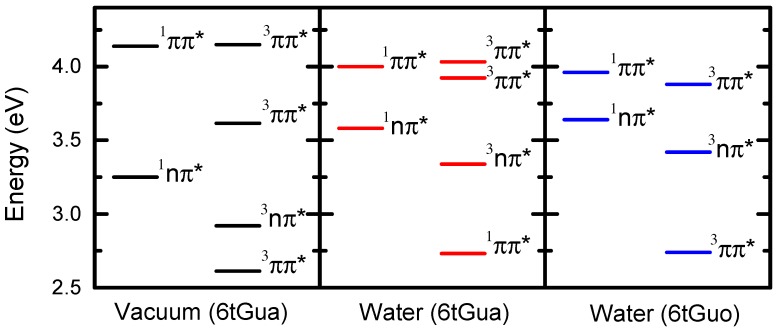
Vertical excitation energies for 6tGua in vacuum and in water environments, and for 6tGuo in water, calculated at the TD-PBE0/IEFPCM/6-311++G(d,p) level of theory. 6tGuo values were reported previously [26].

Ground-state optimizations of 6tGua were performed in vacuum and in water environments at the PBE0/IEFPCM/6-311++G(d,p) level of theory. Vertical excitation energy calculations were performed subsequently on the optimized geometry in the corresponding solvent environment. A reaction field was used to model the solvation effects from water. We note, however, that it has been shown recently that explicit solvent effects are needed to most accurately describe the excitation energies in DNA bases and their thio-derivatives [43,44,45]. The calculations shown in Table 1, and in Figure 2 and Figure 4, indicate that a minimum of five excited-states must be considered, at the TD-DFT level of theory, to explain the relaxation mechanism of 6tGua following excitation with UVA radiation at 345 nm. Particularly, upon excitation, the S_2_(ππ*) state is expected to be populated overwhelmingly (Figure 2) due to its relatively high oscillator strength. Vertical excitations were calculated previously for 6tGua using multi-state second-order perturbation theory on state-average complete active space self-consistent-field wavefunctions (MS-CASPT2//SA-CASSCF) with the ANO-L basis set [46]. The singlet energies computed at this level of theory resemble those from TD-DFT, but the triplet energies are generally much higher in energy (Table 1). This is consistent with the observation that TD-DFT usually underestimate the energies of low-lying triplet states of molecules containing large aromatic systems [47,48]. 

The observation of phosphorescence, and the lack of fluorescence from 6tGua (Figure 2), suggest that a significant portion of the excited-state population undergoes intersystem crossing (ISC) to the triplet manifold, as previously reported for other sulfur-substituted DNA bases [19,26,28,34,49,50]. According to the TD-DFT calculations, intersystem crossing from either the S_2_(ππ*) state to T_2_(nπ*) state, or from the S_1_(nπ*) state to T_1_(ππ*) state, is favorable, as the change in state character is in agreement with the El-Sayed propensity rules (Figure 4) [51]. These pathways are in agreement with those predicted from previous vertical excitation energies computed for 6tGua at the MS-CASPT2//CASSCF(14,12)/ANO-L level of theory in vacuum [46]. 

The TD-DFT calculations performed in this work can be compared directly with those computed previously for the *anti*-sugar conformation of 6tGuo at the TD-PBE0/IEFPCM/6-311++G(d,p) level of theory [26]. As depicted in Figure 4, the S_2_(ππ*), S_1_(nπ*), T_3_(ππ*), T_2_(nπ*), and T_1_(ππ*) vertical excitation energies of 6tGua are all within 0.1 eV of those computed for 6tGuo. This similarity leads to nearly identical energy gaps between the S_2_(ππ*) and T_3_(ππ*), S_2_(ππ*) and S_1_(nπ*), S_2_(ππ*) and T_2_(nπ*), and the S_1_(nπ*) and T_1_(ππ*) states. 

### 2.4. Transient Absorption Spectroscopy of 6tGua

**Figure 5:**
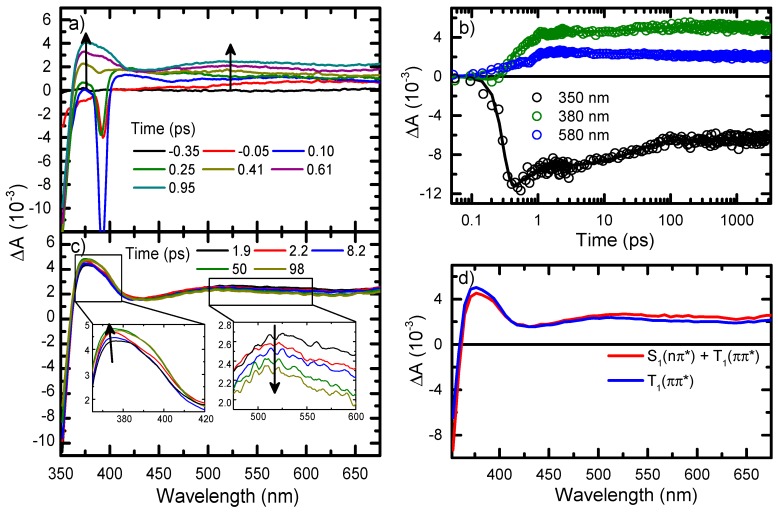
Transient absorption spectra of 6tGua in PBS from (**a**) sub-picosecond to (**c**) picosecond time-delays following 345 nm excitation. The negative signals from ca. 380 nm to 400 nm (**a**) are due to stimulated Raman emissions from the solvent, and are observed within the cross correlation of the pump and probe beams; (**b**) Representative kinetic decay traces of 6tGua in PBS out to 3 ns at indicated probe wavelengths; (**d**) Decay-associated spectra of 6tGua in PBS generated from global analysis using a sequential model.

Figure 5 shows the transient absorption spectra of 6tGua in PBS from sub-picosecond to a time delay of 100 ps. UVA excitation results in the formation of bands centered at 375 nm and 520 nm, as well as a negative-amplitude absorption band at wavelengths shorter than ~360 nm. This absorption band overlaps the ground-state absorption (Figure 2), and thus is assigned to ground-state depopulation. Ground-state depopulation is followed by a slight decay and rise of the 520 nm and 375 nm maxima, respectively, which is observed within 100 ps. During this process, the negative-amplitude absorption band significantly decreases in magnitude (Figure 5b). The resulting transient absorption spectrum remains unchanged through the full 3 ns time-delay available with our delay stage, indicating that the majority of excited-state population decays on nanosecond to microsecond timescales. 

The femtosecond transient absorption kinetics of 6tGua were globally fitted to a two-lifetime exponential model (Figure 5b). Similar lifetimes (within the error) were obtained using a two-component sequential model. Decay-associated spectra generated through global analysis using a sequential model are shown in Figure 5d, and match nicely with the experimental transient absorption spectra. 

**Figure 6:**
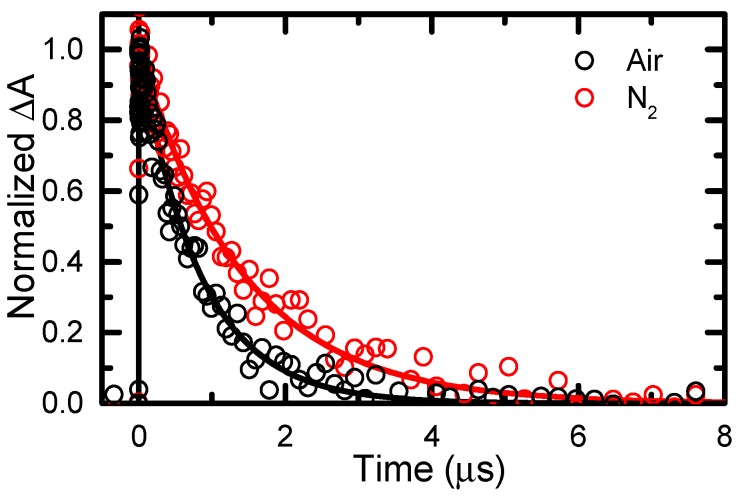
Representative decay-traces for 6tGua triplet absorption monitored at 350 nm in PBS under air- and N_2_-saturated conditions following 345 nm excitation. Traces were fitted to a single-lifetime exponential kinetic model.

The full excited-state decay of 6tGua was monitored using nanosecond transient absorption spectroscopy (~400 ps instrument response function) in air and in deaerated solution (Figure 6). The time-domain transient absorption data was fitted successfully to a single-lifetime exponential model, producing the lifetimes shown in Table 2. The long-lived nature of this excited-state species further supports its assignment to the lowest-energy triplet state. The decay of the T_1_ state is significantly quenched in the presence of molecular oxygen (Figure 6), suggesting that it may undergo reactions to produce reactive oxygen species, as observed recently for 6tGuo [19]. 

**Figure 7:**
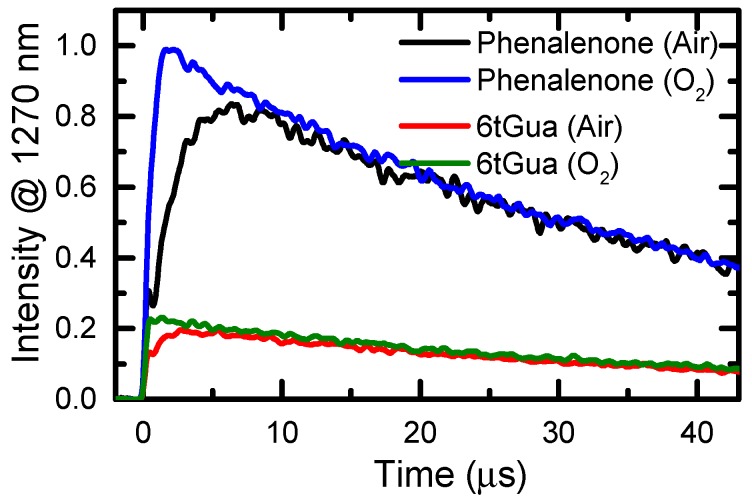
^1^O_2_ phosphorescence signal measured at 1270 nm from air- and O_2_-saturated solutions of 6tGua and the standard phenalenone (Φ_Δ_ = 0.98) [52] in Tris buffer at pH 7.4 following pulsed excitation at 355 nm (7 ns pulse width).

Time-resolved luminescence spectroscopy was employed to quantify singlet oxygen generation from 6tGua following UVA excitation in air- and in O_2_-saturated aqueous solution (Figure 7). As reported in Table 2, the ^1^O_2_ quantum yields are much larger than for the canonical base guanine [20], and are nearly identical in air- and in O_2_-saturated conditions. This is likely due to the relatively long triplet decay lifetime of 6tGua. Previously, Gao et al. measured a ^1^O_2_ quantum yield of 0.56 ± 0.18 for 6tGua following UVA excitation in O_2_-saturated pH 7.4 Tris buffer [9]. As recently discussed in detail for 6tGuo [19], this >2-fold increase compared to the value reported herein is likely a result of an empirical correction factor of 2 applied toward their quantum yield in an attempt to account for oxidation of 6tGua that was observed in their experiments [9]. However, the ^1^O_2_ yield determined by Gao et al. is within error of that reported in this work if this correction factor is not included. 

### 2.5. Excited-State Relaxation Mechanism of 6tGua

The first lifetime (τ_1_) in the transient absorption data of 6tGua corresponds to the rise of the maxima at 375 nm and 520 nm, while the second (τ_2_) corresponds to the decay and rise of the 520 nm and 375 nm bands, respectively. In addition, the negative signal at ca. 350 nm appears to repopulate slightly during τ_1_, but this may result from an increase in excited-state absorption on top of the depopulated region. The most negative ground-state depopulation amplitude (−12 mOD) occurs at a 410 fs time delay (Figure 5), at which point the 375 nm maximum is at an amplitude of 2 mOD. During τ_1_, both the depopulation band (~350 nm) and maximum at 375 nm increase by ~2 mOD, suggesting that the apparent ground-state repopulation in Figure 6 originates from a rise in excited-state absorption. Therefore, we assign τ_1_ to ultrafast population of the triplet manifold in 6tGua, a process that has been reported for 6tGuo and many other sulfur-substituted DNA derivatives [26,27,28,34,50,53,54,55]. This is further supported by the enhanced spin-orbit between the singlet and triplet manifolds expected upon conjugation of the sulfur atom, which has been reported for several thiobases [45,46,49,55,56,57,58,59]. Under the assumption that the transient absorption cross sections for 6tGua in PBS aqueous solution are similar to those reported previously for 6tGuo [26], and that the experimental conditions were similar, a (≥ 60 ± 20)% ISC yield can be estimated for 6tGua. However, we remark that this is simply a crude lower-limit estimation, which is within errors of the reported triplet yield for 6tGuo [26].

As discussed above, TD-DFT vertical excitation energies (Table 1 and Figure 3) indicate that intersystem crossing from the S_2_(ππ*) state to the T_2_(nπ*) state, or from the S_1_(nπ*) state to the T_1_(ππ*) state, should be most favorable, as they both obey the El-Sayed propensity rules [51]. These predictions agree with vertical excitation energy calculations performed at the MS-CASPT2//CASSCF(14,12)/ANO-L level of theory [46]. However, MS-CASPT2//SA-CASSCF calculations were also performed to determine the singlet/singlet and singlet/triplet crossing points and state minima [46,58]. These computations indicate that a S_2_(ππ*)/S_1_(nπ*) conical intersection is accessible barrierlessly from the Frank-Condon (FC) region of the S_2_(ππ*) state potential energy surface (PES), suggesting that internal conversion from the S_2_(ππ*) state to the S_1_(nπ*) state occurs rapidly (~10 fs). The triplet manifold is populated subsequently, primarily through the dark S_1_(nπ*) state, as has be shown for other sulfur-substituted and canonical DNA bases [46,49,59]. The S_1_ PES exhibits both ππ* and nπ* minima with energies of 3.78 eV and 3.18 eV, respectively, while the T_1_ state PES has two ππ* minima at 3.00 eV ((^3^ππ*)_min2_) and 3.37 eV ((^3^ππ*)_min_) [46]. Nonadiabatic surface-hopping dynamic simulations including spin-orbit coupling suggest that the primary ISC pathway initiates from the nπ* minimum of the S_1_ state, initially populating the T_2_ nπ* minimum and the (^3^ππ*)_min2_, which are isoenergetic [46,58]. Pathways from the S_1_ ππ* minimum to the T_2_(nπ*) state, and from the S_1_ nπ* minimum to (^3^ππ*)_min_, are expected to be minor contributions to the overall ISC dynamics [58]. These calculations are consistent with our experimental results and suggest that we should assign τ_1_ to the combination of internal conversion from the S_2_ state to the S_1_ state, ISC from the S_1_ state to the T_2_ state (or T_1_ state), and internal conversion from the T_2_ state to the T_1_ state. 

The second lifetime (τ_2_) observed in the ultrafast relaxation dynamics of 6tGua is more difficult to assign. During this timeframe, the spectral features are nearly identical to those observed for 6tGuo, in which decay of the 520 nm band coincides with a rise of the 375 nm maximum [26]. For 6tGuo, τ_2_ was assigned to a combination of solvation dynamics in the T_1_(ππ*) state and internal conversion from the S_1_ state to the ground state [26]. The latter was proposed due to apparent ground-state repopulation in the transient absorption data, which is also observed for 6tGua (Figure 5). Just as for τ_1_, this may be due to a rise in excited-state absorption on top of the ground-state depopulation signal (Figure 5). However, this is unlikely because the 375 nm maximum only increases in magnitude by ~0.5 mOD, while the depopulated region increases by ~3 mOD, suggesting that the ground-state repopulation process is mostly independent of overlapping absorption bands. This assignment is supported by MS-CASPT2//SA-CASSCF calculations, which predict an energetically-accessible (ΔE = 0.04 eV) conical intersection between the S_1_ ππ* minimum and the ground-state PES [46,58]. Nonadiabatic surface-hopping dynamics excluding spin-orbit coupling (i.e., only singlet dynamics) predict that internal conversion from the S_1_ state to the ground state would last for tens of picoseconds [58], which agrees with the time constant (τ_2_) obtained from global analysis of the transient absorption data. These calculations support the assignment of τ_2_ to internal conversion from the S_1_ state to the ground state, but they cannot explain the rise in magnitude of the transient absorption band centered at 375 nm. For 6tGuo, τ_2_ was shown to decrease significantly from 80 ± 15 to 32 ± 5 ps in going from PBS to acetonitrile, which is consistent with the assignment of solvent dynamics in the T_1_(ππ*) state on an equal time scale. Therefore, we suggest that τ_2_ in 6tGua corresponds to a combination of internal conversion from the S_1_ state to the ground state and solvent dynamics in the T_1_(ππ*) state, as suggested previously for 6tGuo [26], but further work is warranted. From the amplitudes of the bleaching signals before and after τ_2_, and taking into consideration the rise in absorption of the 375 m band, we estimate an upper limit of 25% for internal conversion to the ground sate. We stress that this is simply an estimation, not a quantitative yield.

### 2.6. Effects of N9-Glycosylation on the Photophysics of 6tGua

While the transient absorption spectral evolution of 6tGua and 6tGuo are nearly identical in aqueous solution, they exhibit significantly different excited-state kinetics. For each, τ_1_ was assigned primarily to the population of the lowest-energy triplet state, but this process is shown to occur nearly twice as fast in 6tGuo (Table 2). An increase in the rate of ISC upon N1-glycosylation of 2-thiothymine has been reported previously [28]. However, the opposite trend is observed for τ_2_, which seems to correspond to a combination of internal conversion from the S_1_ state to the ground state and solvent dynamics in the T_1_ state. These differences suggest that N9-glycosylation of 6tGua enhances intersystem crossing from the singlet to triplet manifold, consequently making ground-state repopulation from the S_1_ state less competitive. However, a comparison of the TD-DFT vertical excitation energies, computed with and without N9-glycosylation (Table 1), indicates that the sugar moiety has only a minor impact on the electronic structure of 6tGua at this level of theory [26]. Most notably, the S_1_/T_2_ and S_1_/T_1_ energy gaps remain 0.22 eV and 0.90 eV for 6tGuo in water, nearly identical to those reported for 6tGua in Table 1. Therefore, the increase in the rates of intersystem crossing is proposed to be due to an increase in the density of vibrational states in the S_1_(nπ*), T_2_(nπ*), and T_1_(ππ*) states upon N9-glycosylation, allowing for more effective vibronic coupling between the singlet and triplet manifolds. 

The most notable differences between 6tGua and 6tGuo dynamics are observed at the nano- and microsecond timescales (Figure 6). The triplet state of 6tGua intersystem crosses to the ground state with a 2-fold longer lifetime than 6tGuo using identical concentrations (0.2 mM) under deaerated conditions [26]. This increase in the magnitude of the triplet-state lifetime has major consequences on excited-state reactivity, as 6tGuo shows a marked decrease in the ^1^O_2_ quantum yield in going from O_2_- to air-saturated aqueous solutions, while that for 6tGua remains constant within the error. A similar decrease in triplet-state lifetime was observed for 2-thiothymine upon N1-glycosylation in deaerated acetonitrile [27], and it was suggested that the increased density of vibrational modes from the deoxyribose group enhances coupling between the T_1_ state and the ground state. At this time, a similar argument can be suggested to explain the enhanced rate of triplet decay for 6tGua upon N9-glycosylation. Further investigation is needed to scrutinize whether this is a general phenomenon observed in other sulfur-substituted nucleobases.

## 3. Materials and Methods 

### 3.1. Chemicals

2-Amino-6-mercaptopurine (6tGua, 97%) was obtained from Sigma Aldrich (St. Louis, MO, USA) and used as received. Solutions of 6tGua were prepared in aqueous phosphate buffer saline (PBS) at pH 7.4. PBS was prepared using 0.480 g of sodium dihydrogen phosphate and 0.354 g of disodium hydrogen phosphate dissolved in 400 mL of ultrapure water and adjusted to pH 7.4 with concentrated sodium hydroxide. 

### 3.2. Steady-State Absorption and Emission Spectra

Room-temperature absorption and emission spectra were recorded using a Cary Bio 100 spectrophotometer (Varian Inc., Palo Alto, CA, USA). Samples were prepared in cuvettes with 1 cm optical path length. Ground-state molar extinction coefficients were measured for 6tGua in PBS by making serial dilutions from a prepared stock solution. 

### 3.3. Steady-State and Time-Resolved Emission Spectra

Steady-state and time-resolved luminescence spectra at 77 K were recorded on a Fluorolog-3 fluorimeter (HORIBA Jobin Yvon, Edison, NJ, USA) using 3 mm (inner diameter) quartz tubes inside a quartz liquid nitrogen dewar. The phosphorescence lifetime at 77 K was measured by multichannel scaling on an OB920 spectrometer (Edinburgh Analytical Instruments, Livingston, UK) in conjunction with a pulsed Xe-lamp.

Room-temperature emission spectra were obtained using a Cary Eclipse spectrofluorimeter (Varian, Inc.). Samples were prepared in cuvettes with 1 cm optical path length. Ground-state molar extinction coefficients were measured for 6tGua in PBS by making serial dilutions from a prepared stock solution. Emission spectra were measured at room temperature using an excitation wavelength of 340 nm under air- and N_2_-saturated conditions. Samples were purged for at least 30 min in septum-top cuvettes prior to data collection. Excitation spectra were recorded at each of the emission maxima under air-saturated conditions. Both the emission and excitation spectra were collected at a scan rate of 20 nm/min with the excitation and emission slit widths set to 5 nm and the photomultiplier tube gain set to 800 V.

### 3.4. Time-Dependent Density Functional Theory Calculations

Quantum-chemical calculations were performed using the Gaussian 09 suite of programs [60]. Ground-state optimizations were performed for 6tGua at the PBE0/IEFPCM/6-311++G(d,p) level of theory in vacuum, and water [61,62,63]. Vertical excitation energies were calculated using the optimized ground-state geometries with the TD-PBE0/IEFPCM/6-311++G(d,p) level of theory [64]. The adiabatic triplet energy was estimated from the difference in energy between the optimized triplet and the optimized ground-state geometries. Optimized triplet geometries was calculated at the UPBE0/IEFPCM/6-311++G(d,p) level of theory. The excited-state character was estimated from visual inspection of the Kohn-Sham orbitals and oscillator strengths. The percentage of single-electron contribution (y%) to the vertical excitation energies was calculated using the following expression as done previously for TD-DFT calculations: [65,66]:

(1) $$y\%=\frac{x_{i}^{2}}{\sum_{i=1}^{n}x_{i}^{2}}\times 100$$

where $x_{i}=$ single-electron transition.

TD-DFT calculations have been shown to satisfactorily model the electronic structure of the DNA nucleobases and nucleosides and their thio-derivatives [26,67,68,69,70]. Bulk solvent dielectric effects on the ground-state geometries and on the excited-state vertical excitations were modeled by including self-consistent reaction field (SCRF) calculations using the polarizable continuum model (PCM) with the integral equation formalism (SCRF = IEFPCM) [71]. The estimated error for the calculation of vertical excitation energies using this methodology is between 0.1 eV to 0.3 eV [67], justifying the shift in the vertical excitation energies by 0.15 eV in Figure 2.

### 3.5. Transient Absorption Spectroscopy

Femtosecond broadband transient absorption spectroscopy was employed to study the excited-state dynamics of 6tGua. The laser and spectrometer setup is described in significant detail in previous publications [72,73]. In brief, a Ti:Sapphire oscillator (Vitesse, Coherent, Santa Clara, CA, USA) seeds a chirped pulse regenerative amplifier (Coherent Libra-HE). The amplifier generates 4 mJ, 100 fs pulses at 800 nm with a 1 kHz repetition rate, which are used to pump an optical parametric amplifier (OPA, TOPAS, Quantronix/Light Conversion, Vilnius, Lithuania). A small fraction of the pump beam is split off prior to the OPA and focused through a constantly-moving 3 mm CaF_2_ crystal to generate a broadband probe pulse (320 nm–710 nm). For each molecule, the OPA was tuned to excitation wavelength of 345 nm. Excitation pulses were set to an intensity of 1 µJ at the sample using a neutral density optical filter in order to minimize cross-phase modulation effects as well as sample degradation [65]. The setup employs a mechanical delay stage to adjust the time it takes the probe pulse to reach the sample up to a maximum delay of 3 ns. The broadband probe pulses are split into two fractions. One beam passes through the sample (2 mm path length) to probe the transient signals, while the other serves to correct for changes in the white light continuum throughout the experiment. The pulses are focused into optical fibers leading to CMOS detection units. Generation of the reference signal comes via a chopper wheel, which blocks every other pump pulse, providing an alternating sequence of spectra with and without sample excitation, and thus providing ΔA transient absorption data. In order to observe dynamics at longer than the 3 ns capability of our mechanical delay stage, an electronically-triggered white light source (Eos, Ultrafast Systems, LLC, Sarasota, FL, USA) is used to generate a probe pulse with a spectral window from ~375 nm to 800 nm, a time resolution of 400 ps, and a temporal window of up to 120 μs [72,73]. During the experiments, solutions were continuously stirred using a Teflon stir bar. Samples were prepared at a ground-state absorption of ~0.5 OD (0.2 mM in PBS) at the excitation wavelength for all experiments, unless stated otherwise. When using the Eos white light source, 6tGua was studied in both air- and N_2_-saturated solutions. N_2_-saturated solutions were purged for 30 min prior and blanketed with N_2_ for the course of the experiment. Degradation of 6tGua was monitored at 340 nm in PBS. Samples were replaced with fresh solutions before the change in absorbance reached 5%. 

A homemade software (Labview, National Instruments, Inc., Austin, TX, USA) was used for data collection. Prior to analysis, the data was corrected for group velocity dispersion of the probe pulse using the two-photon absorption signal of neat methanol. Approximately 80 kinetic traces were extracted from the multidimensional transient absorption data for global and target analysis with Igor Pro 6.36 [74]. Traces for 6tGua were globally fit to a two-lifetime exponential model [75]. The instrument response function was held at 200 fs in all cases, as determined from the two-photon coherent signal from neat methanol (IRF of 200 ± 50 fs) [76]. The nanosecond decay dynamics of 6tGua were collected using the Eos white light source and 30 kinetic traces were extracted from each data set and globally fit to a single-exponential kinetic model. 

### 3.6. Determination of Singlet Oxygen Yields

Nanosecond time-resolved luminescence spectroscopy was employed to determine the quantum yield of ^1^O_2_ produced by 6tGua following UVA excitation [19,28,54]. Briefly, a GCR-150-30 Nd:YAG laser (Spectra Physics, Santa Clara, CA, USA, 355 nm, 7 ns pulse width) was used as the excitation source. ^1^O_2_ phosphorescent decay traces were collected at 1270 nm using a modified Fluorolog-3 spectrometer (HORIBA, Jobin Yvon) with a NIR sensitive photomultiplier tube (H102330A-45, Hamamatsu, Hamamatsu City, Japan). The decay traces were then stored on a digital oscilloscope (TDS 360, Tektronics, Beaverton, OR, USA). Solutions of 6tGua and the phenalenone standard were prepared in Tris-buffered D_2_O at an optical density of 0.3 at 355 nm in 1 cm path length quartz cuvettes. The O_2_-saturated solutions were bubbled with ultrapure oxygen gas for >20 min prior to testing. Degradation of the samples was determined to be less than 3% over the course of the experiments based on their steady-state absorption spectra. The quantum yields were determined in back-to-back luminescence experiments of 6tGua and phenalenone solutions under identical conditions, using the reported yield of ^1^O_2_ generated by phenalenone (Φ_Δ_ = 0.98) [52]. 

## 4. Conclusions

The excited-state dynamics of 6tGua have been studied from femtosecond to microsecond time-delays following UVA excitation under physiological conditions. TD-DFT computations and previous high-level quantum-chemical calculations for 6tGua were used to assist in the assignment of the relaxation pathways. We show that 6tGua populates the triplet manifold within ~600 fs followed by decay of the excited-state population from the lowest-energy triplet state to the ground-state in 1.4 and 0.8 μs in deaerated and in air-saturated aqueous solution, respectively. Upon UVA excitation, 6tGua produces ^1^O_2_ with quantum yields of 0.21 (air) and 0.23 (O_2_) in PBS, indicating that the nucleobase has the potential to act as Type II photosensitizer before metabolization in the cell cytoplasm. These yields are in good agreement with those reported recently for 6tGuo [19], but approximately twofold less than those previously reported by Gao et al. for 6tGua [9]. They are also twice as small as those reported recently for mono- and di-sulfur substituted pyrimidine bases under equal experimental conditions [28,54].

To provide insight into the effects of N9-glycosylation on the excited-state dynamics of 6tGua, we compared our results with those previously reported for 6tGuo under similar conditions. Notably, 6tGuo populates the triplet manifold nearly twice as fast as 6tGua following UVA excitation. The same effect was recently reported for 2tThy, in which N1-glycosylation decreases the magnitude of the ISC lifetime by ~200 fs [28]. Additionally, intersystem crossing from the lowest-energy triplet-state to the ground state occurs nearly twice as fast for 6tGuo in deaerated aqueous solution. A comparison of TD-DFT calculations including solvent effects indicates that N9-glycoslyation should have minimal effects on the electronic structure of 6tGua [26]. However, an increased density of vibrational modes expected upon glycosylation could enhance vibronic coupling between the singlet and triplet manifolds. This can explain the increased rate of ISC observed for 6tGua following glycosylation, and is supported by a recent comparison of 2-thiothymine and 2-thiothymidine in acetonitrile, in which 2-thiothymidine exhibited a significantly shorter triplet lifetime [27]. Therefore, the results presented in this work contribute significantly to understanding and generalizing the role of glycosylation on the photochemical properties of these prodrugs, as well as on other thiobase derivatives. 

## Figures and Tables

**Table 1:** Vertical excitation energies for 6tGua in vacuum and in water environments, and for 6tGuo in water [26], computed at the TD-PBE0/IEFPCM/6-311++G(d,p) level of theory. Oscillator strengths are provided in parentheses. Vertical excitation energies computed at the MS-CASPT2//CASSCF(14,12)/ANO-L level of theory [46] are also given for comparison.

State	Vacuum (eV)	Vacuum (eV) [46]	Water (eV)	6tGuo Water (eV) [26]
S_2_(ππ*)	4.14 (0.26)	4.05 (0.54)	4.00 (0.40)	3.96 (0.45)
S_1_ (nπ*)	3.25 (0.00)	3.36 (0.00)	3.58 (0.00)	3.64 (0.00)
T_3_ (ππ*) ^a^	3.62	4.24	3.92	3.88
T_2_ (nπ*)	2.92	3.31	3.34	3.42
T_1_ (ππ*)	2.61	3.10	2.73	2.74
ΔE(S_2_-S_1_)	0.89	0.69	0.42	0.32
ΔE(S_2_-T_3_)	0.52	−0.19	0.08	0.08
ΔE(S_2_-T_2_)	1.22	0.74	0.66	0.54
ΔE(S_1_-T_2_)	0.33	0.05	0.24	0.22
ΔE(S_1_-T_1_)	0.64	0.26	0.85	0.90

^a^ The T_3_ state of 6tGuo was previously reported as having primarily nπ* character in [26]. Re-examination of the vertical excitation energies calculations for 6tGuo in water reveals instead that the T_3_ state has mostly ππ* character.

**Table 2:** Lifetimes of 6tGua extracted from global fitting of transient absorption data. Also included are those reported for 6tGuo in PBS pH 7.4 [19,26].

Base	τ_1_ (ps)	τ_2_ (ps)	τ_3_ (ns) (Air)	τ_3_ (ns) (N_2_)	Φ_Δ_ (Air)	Φ_Δ_ (O_2_)
6tGua	0.56 ± 0.06	26 ± 3	830 ± 70	1420 ± 180	0.21 ± 0.02	0.23 ± 0.02
6tGuo	0.31 ± 0.05 [26]	80 ± 15 [26]	460 ± 15 [26]	720 ± 10 [26]	0.13 ± 0.02 [19]	0.24 ± 0.02 [19]

## Supplementary Materials

The following are available online. Figure S1: (a) Emission spectra of 6tGua in PBS with an excitation wavelength of 340 nm under air- and N2-saturated conditions. (b) Excitation spectra at the 420 nm and 480 nm emission maxima is plotted with the ground-state absorption spectrum of 6tGua, Figure S2: Kohn-Sham orbitals of 6tGua associated with the single-electron transitions of relevant excited-states with energies equal or less than the 345 nm excitation used in this work.
