# Supplementary material for: Excited-State Dynamics of the Thiopurine Prodrug 6-Thioguanine: Can N9-Glycosylation Affect Its Phototoxic Activity?
Source: Molecules. 2017 Feb 28;22(3):379. doi: 10.3390/molecules22030379 (PMC6155220; doi:10.3390/molecules22030379)
Supplement: Supplementary file 1 [file molecules-22-00379-s001.pdf]

Excited-State Dynamics of the Thiopurine Prodrug 6-Thioguanine: Can N9-Glycosylation Affect Its Phototoxic Activity?

Brennan Ashwood [1], Steffen Jockusch [2], and Carlos E. Crespo-Hernández [1,*]

[1] Department of Chemistry and Center of Chemical Dynamics, Case Western Reserve University, Cleveland, Ohio 44106, United States
[2] Department of Chemistry, Columbia University, New York, New York 10027, United States
* Correspondence: carlos.crespo@case.edu

Supporting Information

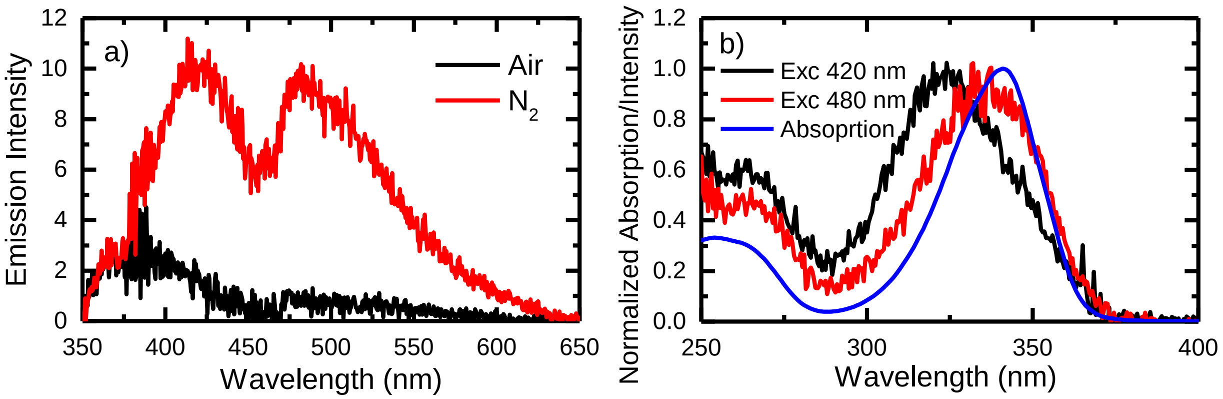

Figure S1. (a) Emission spectra of 6tGua in PBS with an excitation wavelength of 340 nm under air- and N_2-saturated conditions. (b) Excitation spectra at the 420 nm and 480 nm emission maxima is plotted with the ground-state absorption spectrum of 6tGua.

Steady-state emission of 6tGua was monitored using an excitation wavelength of 340 nm (Figure 2a). Under air-saturated conditions, the emission spectrum exhibits maxima at 385 nm and ~ 480 nm. Using an identical concentration of 6tGua under deaerated conditions, a third emission band from 375 nm to 450 nm with maximum at 420 nm is produced, and the emission band at 480 nm exhibits a 10-fold increase in emission intensity.

Excitation spectra were obtained at 420 nm and 480 nm (Figure S2b). The excitation spectra using 480 nm emission is nearly identical to the ground-state absorption spectrum of 6tGua and that from 420 nm emission is blueshifted approximately 15 nm from that of the ground-state absorption. This suggests that the emission band at 420 nm comes from a photoproduct or impurity and that centered at 480 nm is from 6tGua. Previously, a similar emission band from 6tGua centered near 410 nm was assigned to fluorescence from guaninie-6-sulfonate, a promutagenic product of 6tGua following oxidation by $^1O_2$.[1,2] Measurements under air- and N_2-saturated conditions were performed consecutively with the same solution, producing more guanine-6-sulfonate and effectively increasing the magnitude of emission at 420 nm. The observation that the emission band beginning at ~450 nm is significantly redshfited (75 nm or

4400 cm^{-1}) from the lowest-energy absorption of 6tGua (λ_{max} ~375 nm), and is also quenched by molecular oxygen, suggests that the band corresponds to phosphorescence from the lowest-energy triplet state to the ground-state. This is in agreement with the results presented at 77 K.

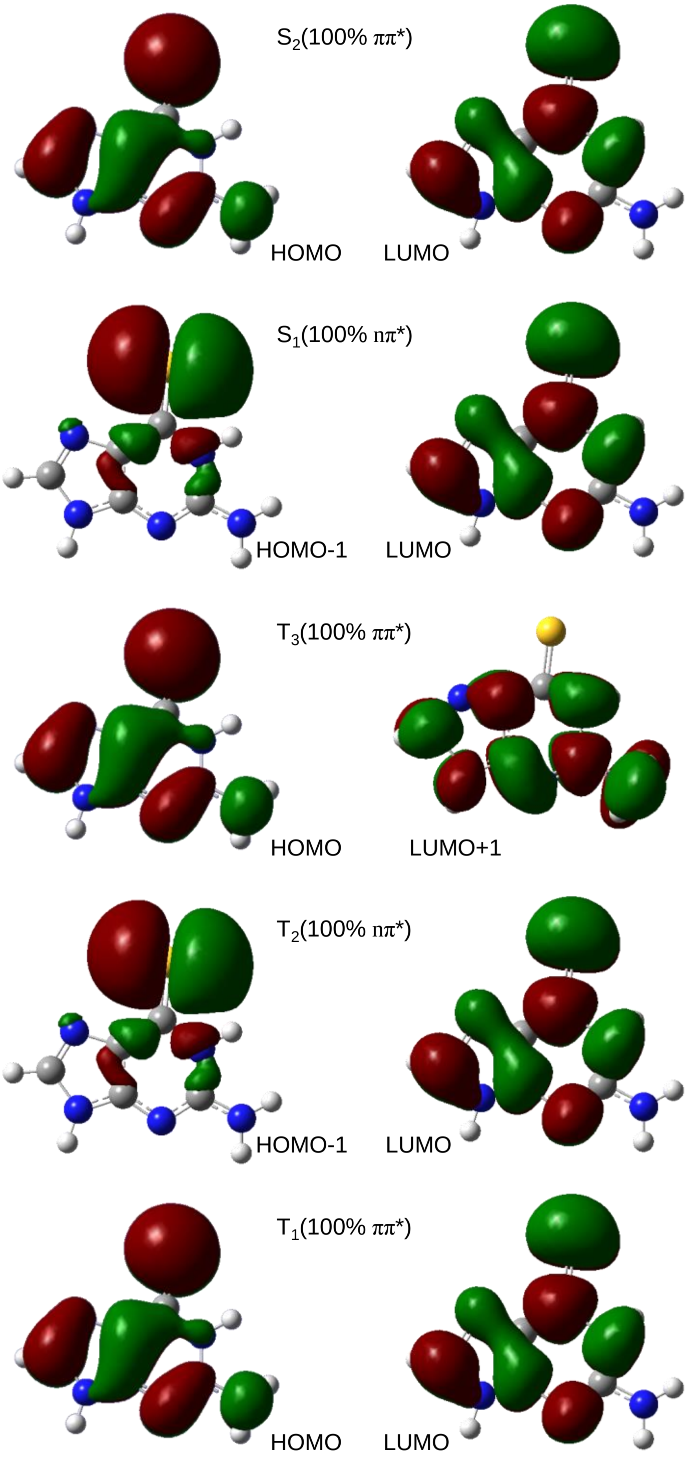

Figure S2. Kohn-Sham orbitals of 6tGua associated with the single-electron transitions of relevant excited-states with energies equal or less than the 345 nm excitation used in this work.

References.

1. Zou, X.; Zhao, H.; Yu, Y.; Su, H. Formation of guanine-6-sulfonate from 6-thioguanine and singlet oxygen: A combined theoretical and experimental study. *J. Am. Chem. Soc.* **2013**, *135*, 4509-4515.
2. Finkel, J.M. Fluorometric assay of thioguanine. *J. Pharm. Sci.* **1975**, *64*, 121-122.
